# The influence of active video game play upon physical activity and screen-based activities in sedentary children

**DOI:** 10.1371/journal.pone.0269057

**Published:** 2022-06-14

**Authors:** Kelsey E. Ufholz, Kyle D. Flack, James N. Roemmich

**Affiliations:** Grand Forks Human Nutrition Research Center, Agricultural Research Service, United States Department of Agriculture, Grand Forks, North Dakota, United States of America; Public Library of Science, UNITED KINGDOM

## Abstract

**Background:**

Few children meet physical activity recommendations, partly due to the prevalence of screen-based sedentary activities. Active video game (AVG) play produces light to moderate physical activity. Yet, providing children access to AVG does not increase physical activity, possibly because children who play AVG may also tend towards sedentary screen-based activities. How multiple days of AVG play influences children’s choice of other activities is not yet known.

**Purpose:**

To examine how AVG influences children’s physical activity, sedentary screen-based activities, and other alternative activities.

**Methods:**

Sedentary children (N = 49) played AVG 3 times/week and sedentary video games (SVG) ad libitum for 6 weeks, followed by 4 weeks of ad libitum play of both AVG and SVG. Participants wore an activity monitor for 7 days and completed a 24-hour activity recall on 4 randomly selected days at baseline, week 6, and week 10.

**Results:**

AVG play increased during the intervention (p < 0.01). Light activity and SVG play both decreased baseline to 10 weeks (p = 0.006) and 6 to 10 weeks (p = 0.017). Non-SVG sedentary behavior increased from baseline to 10 weeks (p = 0.005) and 6 to 10 weeks (p = 0.007). Changes over time were not observed in physical activity, or recall-measured active play, social activities, other hobbies, television or computer/phone use.

**Conclusion:**

AVG play did not change children’s objectively-measured physical activity or subjectively measured active play. SVG time was substituted with other sedentary behaviors. AVG did not increase time engaged in SVG or screen-based devices.

## Introduction

Current public health guidelines recommend that youth engage in physical activity (PA) for at least 60 minutes per day. This may take the form of either unstructured active play or formal exercise, such as sports [1]. Despite best efforts by parents, educators, researchers, and the United States government, in 2017 only 26.1% of adolescents met this criteria [2], contributing to poorer physical and mental health [3, 4] and increased mortality during adulthood [5, 6].

Behavioral Choice Theory [7] states that individuals have a variety of options competing for their leisure time, with the most reinforcing alternative often chosen. Sedentary screen-based activities are particularly pervasive in our environment [8] and more reinforcing than PA, causing youth to spend time engaging in screen-based activities than PA [9, 10]. Among America and Australian adolescents, excessive screen time is associated with reduced PA [11] and correlated with poorer health independent of physical activity [12]. Therefore, to maximize health effects, children should be encouraged to simultaneously reduce sedentary screen-based activities while increasing PA.

A putative method to increase PA in children is by combining highly reinforcing screen-based technology with physical movement in the form of active video games (AVG). Indeed, AVG play facilitates greater energy expenditure and promotes low, moderate, and vigorous PA in children [13–17]. Conflicting research does exist, with one study demonstrating no increased energy expenditure over non-active sedentary video games [18], although this variation may be partly attributed to differences between game types. Games which promote lower body movements, such as kicking and running, tend to produce greater energy expenditure [19]. A recent meta-analysis found that although AVGs did not increase physical activity levels over minimal control conditions in the short term (3 > months), AVG play was more effective in decreasing BMI with longer follow-up (3–12 months) [20]. AVGs also have several advantages over traditional active play (TAP): they are not dependent upon weather and season, can be played at home, individually or in a group, and offer an exercise option for children who are socially anxious, uncoordinated, or have a medical condition or physical disability which hinder TAP [14]. Although AVGs have the disadvantages of being played less intensely and resulting in less energy expenditure than TAP [15], perhaps they facilitate entry into other forms of TAP, by increasing PA self-efficacy [21] and improving gross motor skills [22].

If AVG are not effective at increasing time in other forms of PA, then for AVG to serve as a viable option for PA, children must play them regularly over a long period of time. Unfortunately, despite high initial interest, AVG play declines over time [23]. Therefore, factors relating to sustained interest in and motivation towards children’s AVG engagement are worth exploring. Factors, both internal to the child such as self-efficacy for active play [24] and intrinsic or integrated regulations for PA [25], and externally imposed variables such as parental support for PA [24, 26], have been shown to increase youth’s time spent TAP. Children who perceive high parental support for PA, report greater PA [27]. Among adolescent girls, 12 weeks of dance-based AVG play resulted in increased self-reported PA, as well as improved PA self-efficacy, and high intrinsic regulation towards AVG play [21]. If younger children show similar increases in self-efficacy and intrinsic motivation when they begin AVG play, they may be more likely to maintain AVG play long-term.

Ideally, AVG could be used to either supplement active play or displace sedentary screen time in children. How increased exposure to AVG play impacts children’s choices of other activities is currently unknown. Simply providing children with AVG systems does not increase their overall activity [18]. This suggests AVG play does not encourage other forms of active play and children may even compensate by decreasing time in TAP. Children who play AVG also tend to engage in screen-based sedentary activities, such as playing sedentary video games and watching television [28]. It is possible that, rather than displacing sedentary screen time, AVG play will lead to increased time spent in other screen-based activities. However, we aimed to investigate whether beginning AVG play would unintentionally lead to increased time in in screen-based activities, including SVG.

### Hypotheses

This study aimed to examine of the effect of beginning AVG play on children’s time spent in other pursuits, both physical and sedentary. It was hypothesized that AVG exposure would lead to greater overall PA, with greater increases in PA positively associated with increased PA self-efficacy, increased intrinsic regulation towards PA, and greater parental support for PA. It was further predicted that time spent in TAP and AVG would result in decreased time spent in other activities, such as light PA. Whether time spent in screen-based sedentary activities would increase, decrease, or remain consistent was an open question.

## Materials and methods

### Study design

Study assessments were conducted by trained research staff between November 2016 and April 2018. AVG play, the study intervention, took place in participants’ homes. This study received approval from University of North Dakota’s Institutional Review Board and was registered at ClinicalTrials.gov (NCT02940431). The study was originally conducted as a randomized controlled trial with a two-group factorial design examining higher and lower-autonomy over AVG play on PA reinforcement. Results of the primary and other study outcomes, including whether autonomy over AVG play influences long-term motivation to play AVG have been published previously [29]. Post-hoc power estimates for time spent in MVPA, (p < 0.05, 1-β > 0.80, effect size h^2^ = 0.20–0.45 [15] suggest adequate power. The current analyses are based on all subjects treated as one group.

### Participants

Participants were healthy inactive children (N = 49) (aged 8–12 y). Participants were 51% female (n = 26). The majority identified as white (76.9%, n = 40), with minorities identifying as black (1.9%, n = 1), Asian (7.7%, n = 4), Hawaiian/Pacific Islander (1.9%, n = 1), multiracial (9.6%, n = 5), and Hispanic (1.9%, n = 1). Of the 52 participants who entered the study, one withdrew prior to randomization, two withdrew after randomization, and 49 completed the study. Both non-overweight and overweight children (5^th^-97^th^ BMI percentile) were included (Table 1). Entry criteria included not currently trying to lose weight, no medical conditions which might impede PA, not playing an organized sport or engaging in leisure time moderate to vigorous PA more than three times per week for one hour at a time, not currently playing AVG, using a screen-based device such as a phone or computer on average at least two hours per day, and willingness to adhere to study treatments and measurement schedules. Families which indicated already owning an AVG system were permitted entrance in the study if they confirmed that their child used the AVG system for less than half an hour per week. Participants’ parents learned of the study via online advertisements on the research institute’s website and word of mouth from other participants and their families.

**Table 1:** Baseline demographics.

Age (yrs)	9.9 ± 1.2^a^
Weight (kg)	40.9 ± 13.3^a^
BMI (kg/m^2^)	19.0 ± 3.7^a^
BMI percentile	62.4 ± 28.5^a^
Gender	Male = 46.2%
	(N = 24)
	Female = 53.9%
	(N = 28)

N = 52

Data are mean ± SD

### Procedure

#### Screening and consent visit

Initial screening took place online. The first in-person session included a review of answers to the online screening questions, study procedures, informed consent from the parent, and assent from the child. Parents were asked to provide demographic and health history information regarding their child. Children were measured for height and weight. Children and their parent(s) filled out the first assessment measures with help from the research staff. During later appointments, families could also take questionnaires home to fill out on their own time. Children were also given an accelerometer and instructions on how to wear it.

#### Intervention

Following baseline assessments, families were provided with an Xbox 360^™^ game system with the necessary accessories, including a Kinect^™^ motion detection system which displays the player’s outline on the television screen. Participants also received both AVG (Kinect Dance 2015^™^, Kinect Dance 2016^™^, Kinect Sports Season 1^™^, Kinect Sports Season 2^™^, and Kinect Adventures^™^) and sedentary video game (SVG) discs (Minecraft^™^, Lego Avengers^™^, Lego Star Wars^™^, NHL Legacy^™^ edition, NBA2K16^™^), with each disc containing a range of games and gaming options. AVG and SVG utilized during this study were chosen based upon popularity with the target demographic and age-appropriate ratings for 8-12-year-old children. AVG required active play-like body movements, better to simulate traditional play, such as running, kicking, and dancing. Participants received 1–2 AVG discs and 2 SVG discs, which were exchanged for new discs every 2 weeks. The number of discs received was based upon autonomy groups children were randomized into. Groups were block randomized based on participants’ gender, BMI, and baseline liking of AVG [29]. These games increase energy expenditure compared to sedentary video games [30, 31] and have been commonly used in studies of energy expenditure, physical activity, and similar topics in this age-group [30, 31]. Similar matching methodologies have been used in prior studies, both in adults and children, examining competing choices between physical activity and sedentary activity [15, 32].

Participants were given AVG discs and were instructed to play the games three times per week. Number and chose of AVG varied based upon which group participants were randomized into and are detailed in a prior publication [29]. Participants could play SVG as they chose but could not play any video games except those provided by the research team. Parents were instructed to not place limits on their children playing SVG. Every two weeks, participants received a new set of video game discs. Participants were instructed to record their video game playing time on a log sheet, which parents verified. AVG games could be played individually or in pairs and participants were encouraged to share their play with friends and family members. The research team was available to install the game system in participants’ homes upon request. Previously published results indicated that children rated their enjoyment of both AVG and SVG at 7 to 9 on a 10-point Likert scale and their enjoyment did not decrease from baseline to 6 and 10 weeks [29].

Following six weeks of treatment, children completed the post-intervention assessments and entered a four-week washout period. During washout, participants were supplied with two AVG and two SVG of their choosing. Children could play both game types *ad libitum*, and any other video games that they might already have at home. After four weeks of washout, children completed their final assessments, identical to those at baseline and 6 weeks. Following the assessment, participants were debriefed, returned the Xbox console and games, asked for intervention feedback, and thanked for their time. The child received $435 and the parent $110 as compensation.

### Measurements

#### Height and weight

Body weight was measured via a Tanita scale. Participants wore light clothing and no shoes during measurement. Height was measured using a stadiometer. BMI percentile was calculated based upon growth curves for each participant’s age and gender [33].

#### Usual physical and sedentary activity

Physical activity was assessed with the ActiGraph WGT3X-BT accelerometer. Children were instructed to put the accelerometer on upon waking and only take it off while sleeping or in water-based activities. Parents were instructed regarding the importance that their child wore the accelerometer at least 10 hours/day, including during AVG play for 7 consecutive days during the indicated study periods. Parents also received written instructions on use, including appropriate care and placement on the right iliac crest [34]. Actigraph data was carefully screened and if insufficient wear time was noted, the monitor was returned to the participant and they were asked to wear it until enough wear time was logged. A minimum of 7 days of valid data was required for analysis. Compliance rates came to 85.7%. Data were cleaned of non-wear time, defined as consecutive strings of zeros greater than 20 minutes, and consolidated into 10 second epochs. Data provided measurements of raw acceleration, energy expenditure, MET rates, and minutes and number of bouts lasting at least one minute spent at different levels of physical activity intensity (sedentary, light, moderate, vigorous, very vigorous), as defined by count thresholds developed by Evenson et al. [35, 36]. ActiGraph accelerometers has been extensively used in research studies and validated for use in children ages 8–12 [36–38]. Participants were instructed to wear the activity monitors at baseline, 6 weeks, 10 weeks, and twice during the intervention itself, as a check for study compliance.

#### 24-Hour activity recall

As a check on compliance and method of quantifying minutes of play, children were instructed to fill-out a recall measure describing the previous day’s activities. An automated message system emailed parents links to an online recall questionnaire on two randomly weekdays and two weekend days. Recalls were completed at baseline, at weeks 2 and 4 of the intervention, after the intervention (6 weeks), and after washout (10 weeks). The children were asked to report hours and minutes spent in the following activities: AVG, SVG, TAP (physically active games / sports that do not involve screens), school, chores, traveling by car/bus, other hobbies (practicing piano, reading for fun), social activities (spending time with friends and family), watching TV/DVD, using a computer/phone etc. The recall was based upon a recall diary used to measure AVG play in adolescents [28]. Similar recall measures, paired with objectively measured PA, have shown valid results in children and adolescents [39]. To ensure compliance, the study team remotely monitored when the measure was completed and sent frequent reminders. Children were allowed to select new video games only after the recall measures were completed. Parents helped their children complete the measure, as needed.

#### Exercise self-efficacy

Participant’s self-efficacy was measured using the Children’s Self-Perceptions of Adequacy in and Predilection of Physical Activity Scale (CSAPPA). This scale has three subscales which measure the child’s beliefs about their ability to perform well in physical activities such as physical education classes and sports teams (adequacy), their likelihood to choose PA over a sedentary activity (predilection), and enjoyment of PA. The scale consists of 20 items which children rate as “really true for me” or “sort of true for me” [40]. The scale was designed for use with children ages 8–16 and has been successfully used experiments with similar age groups [15]. The scale has been shown to have adequate test-retest reliability as well as predictive validity [40], and excellent internal consistency (α ≥ 0.89) [41].

#### Motivation for physical activity

Motivation for PA was measured with the Behavioral Regulations in Exercise Questionnaire, 2^nd^ edition (BREQ-2). The original BREQ was developed as a measure the self-determination theory’s continuum of external vs. internal motivation for PA (“I exercise because it’s fun”) [42] and examined four subscales: external regulation, introjected regulation, identified regulation and intrinsic regulation. The BREQ-2 includes an additional subscale measuring amotivation [43]. The BREQ has good psychometric properties with children ages 7–11 [44] has been utilized successfully with children ages 8–11 years [45].

#### Motivation for Active Video Games (AVG)

Questions from the BREQ-2 [43] were modified so that the question refers to motivation towards AVG rather than PA (“I play active video games because it’s fun”). While not specifically designed for alternate behaviors, variations of the BREQ-2 have been successfully used to measure motivations for non-exercise behaviors such as children’s oral health [46].

#### Motivation for Sedentary Video Games (SVG)

Questions from the BREQ-2 [43] were also modified so that the question refers to motivation towards sedentary video games behavior rather than PA (“I play video games because it’s fun.”).

#### Physical activity-related parenting behaviors

Parents filled out The Activity Support Scale for Multiple Groups (ACTS-MG) developed by Davison (2011) [47]. The ACTS-MG includes 12 items, 3 each in: Logistic Support (parents making provisions enabling their child to be physically active), Explicit Modeling (parents using their own behavior to encourage their child to be active), Use of Community Resources (make use of community resources to get their child to be active) and Restricting Access to Sedentary Activities. Cronbach’s alpha ranges from 0.69 to 0.88. Items are measured on a 4-point scale (0 = strongly disagree; 4 = strongly agree) with higher scores showing greater parental support for PA. The scale has good overall factorial validity and internal consistency [47].

### Analytic plan

Preliminary analyses examined data for normality, including skewness and kurtosis. Values which were found to meet the definition of an outlier [48] were excluded. Of those who completed the study, three were found to have objectively measured baseline PA exceeding the entry criterion. Those subjects’ data were excluded from subsequent analyses. AVG, SVG, and TAP recall data showed notable skew and many data were zero, so these were transformed by adding one and taking the log base 10 value. Children were instructed to fill out the psychological questionnaires at all three timepoints (baseline, 6 weeks, 10 weeks). Questionnaires filled out by the parents were only completed at baseline.

The main outcome consisted of minutes spent in moderate to vigorous PA (MVPA), light PA, and sedentary activity from the accelerometer, number of bouts per day of light PA and MVPA, and recall data for minutes per day of AVG, SVG, TAP, computer/phone, TV/DVD, other hobbies, and social activities. MVPA was analyzed via restricted maximum likelihood mixed models with phase (baseline, 6 weeks, 10 weeks), as the within subjects’ factor. To statistically account for autonomy over AVG play treatment group, autonomy group was included as a covariate in the analyses. Other covariates included as control variables were baseline MVPA and total activity monitor wear time. Parallel analyses were run with light PA, sedentary time, bouts of MVPA, and bouts of light PA as outcome variables, with the corresponding baseline values as covariates. To account for multiple comparisons, Tuker-Kramer contrasts were utilized.

To examine the association between time spent in physical or sedentary activity (MVPA, light PA, sedentary time, light activity bouts, MVPA bouts, minutes per week of TAP, AVG, and SVG) and psychological constructs (Enjoyment, Predilection, and Adequacy, intrinsic, external, introjected, integrated, identified regulation, amotivation, parental Logistic Support, Modeling, Community Resources, and Restricting Sedentary), a series of Pearson correlations were performed. Correlations were performed between delta scores (6 week or 10-week scores minus baseline scores) with the exception of those utilizing subscales of the ACTS-MG, Logistic Support, Modeling, Community Resources, and Restricting Sedentary. Correlations were interpreted according to the guidelines for Pearson correlations in psychology in Akoglu [49] as strong (r = 0.9–0.7), moderate (r = 0.40–0.69), weak (r = 0.10–0.39), and zero (r < 0.10).

Validity of the BREQ-AVG and SVG measures were established via inter-measure subscale correlations. These validity correlations were with 6-week subscale total scores, to ensure all participants were drawing on experience with AVG and SVG when providing answers. Internal validity of questionnaire subscales was conducted via Cronbach’s alpha. Internal consistencies of the novel BREQ subscales were comparable to (BREQ-AVG α = 0.54–0.91) or exceeded (BREQ-SVG α = 0.82–0.90) that of the BREQ-PA (α = 0.59–0.83). Most of these were in the range considered in the acceptable range (α = 0.7–0.95) [50]. Sample size was determined a priori based upon estimated effect sizes for PA reinforcement [29]. All analyses were performed on SAS 9.4 or SPSS 26.

## Results

### Physical activity

No effect of time was found for MVPA (p = 0.092). There was a significant (p = 0.015) effect of time for light activity. Specifically, light activity decreased from baseline to 10 weeks (p = 0.020) and 6 to 10 weeks (p = 0.050). There was a significant effect of time (p = 0.024) for sedentary activity. Specifically, sedentary behavior increased from baseline to 10 weeks (p = 0.025). No effect was of time was found for bouts of light activity (p = 0.411) or bouts of MVPA (p = 0.251) (Table 2).

**Table 2:** Minutes and bouts per day spent in moderate to vigorous, light and sedentary activity by time point.

	Baseline	6 weeks	10 weeks
MVPA	27.01 ± 2.1	28.19 ± 2.1	23.08 ± 1.6
Light Activity^a^	343.80 ± 8.9*	341.92 ± 7.9^#^	326.46 ± 9.0*^,^^#^
Sedentary Activity^a^	409.18 ± 13.0*	415.65 ± 9.9	431.31 ± 10.4*
Bouts light Activity	2.32 ± 0.2	2.21 ± 0.2	2.81 ± 0.7
Bouts MVPA	3.45 ± 0.3	3.54 ± 0.3	3.24 ± 0.3

MVPA = minutes per day spent in moderate to vigorous PA

Light Activity = minutes per day spent in light PA

Sedentary Activity = minutes per day spent in sedentary activity

Bouts light activity = Bouts per day of light PA lasting at least 1 minute

Bouts MVPA = Bouts per day of moderate to vigorous PA lasting at least 1 minute

Data are mean and standard error

^a^time effect p<0.05.

*, ^#^ Means with like symbols are different p<0.05.

### AVG, SVG, TAP

There was a significant effect of time for AVG play (p < 0.001); specifically, AVG play time increased from baseline to 6 weeks (p < 0.001) and decreased from 6 to 10 weeks (p < 0.001). There was a significant effect of time for SVG play (p < 0.001); SVG play decreased from baseline to 6 weeks (p = 0.001) and baseline to 10 weeks (p < 0.0001). There was no effect of time for TAP (p = 0.238) (Table 3).

**Table 3:** Minutes per day spent in active video games, sedentary video games, television, computers, other hobbies and social activities by time point.

	Baseline	6 weeks	10 weeks
AVG^a^	2.39 ± 0.9*	16.61 ± 2.8*^#^	6.63 ± 1.9^#^
SVG^a^	49.30 ± 8.2*^#^	28.52 ± 6.1*	28.06 ± 6.2^#^
TAP	35.73 ± 5.0	32.21 ± 5.5	34.67 ± 5.1
TV	92.26 ± 8.5	82.88 ± 9.4	93.99 ± 10.4
Computer^a^	38.01 ± 5.7	30.34 ± 6.7	40.09 ± 7.7
Hobbies	55.03 ± 5.8	45.59 ± 6.2	50.34 ± 6.7
Social	178.80 ± 16.1	168.26 ± 16.5	182.40 ± 19.2

Data are mean and standard error

AVG, SVG, TAP and computer are untransformed means

AVG = minutes per day spent playing active video games

SVG = minutes per day spent playing sedentary video games

TAP = minutes per day spent playing traditional active games

^a^time effect p<0.05

*, ^#^ Means with like symbols are different p<0.05.

### Other activities

Computer/phone time showed notable skew, so these data were log base 10 transformed. Backtransformed data are presented in Table 3. Computer/phone use showed no effect of time (p = 0.071). Computer showed an overall effect of time (p = 0.034) with the specific time effect of baseline to 6 weeks at, p = 0.053.

There was no effect of time for social activities (p = 0.614), TV (p = 0.312), and other hobbies (p = 0.353).

### Seasonal impacts upon AVG play

Ad hoc analyses were run to determine whether AVG play varied depending upon season and the school year. For these analyses, time effect was replaced by effect of season or school year. Because of the extreme climate of Grand Forks, ND where the study took place, winter was defined as beginning the first week that the average high temperatures did not rise above freezing (32* F) and ending the last week with similar temperatures. This corresponded to the first full week of November and the first full week of April in 2016–2018. Summer was defined as June, July and August, autumn as September, October and the first several days of November, and spring as later April and May. School was considered in session during September through May and out of session during the summer months. Season and school year were substituted as the within subjects’ factor in two separate mixed models similar to the major analyses. AVG play and autonomy were included as covariates. Log base 10 plus 1 transformed values for AVG play were used as the dependent variable. AVG play did not vary by season (p = 0.427) or by school year (p = 0.700).

As shown in Tables 4 and 5, weak positive associations were found at 6 weeks between changes in sedentary behavior and Predilection (p = 0.050). Weak positive association were also found for changes in AVG play and both identified (p = 0.049) and introjected regulation (p = 0.047). SVG play weakly positively associated with parent’s use of community resources (p = 0.008) and restriction of sedentary activities (p = 0.015). TAP and identified regulation also showed a weak positive association (p = 0.027). At 10 weeks, weak positive associations were found between light PA and parental use of community resources (p = 0.023). Similar weak positive associations were found between AVG play and external regulation (p = 0.025). SVG play weakly, positively associated with parent’s modeling of physical activity (p = 0.020), use of community resources (p = 0.016), and restriction of sedentary activities (p = 0.011). TAP weakly, positively associated with parent’s logistic support of physical activity (p = 0.006).

**Table 4:** Univariate correlations of child physical activity delta scores, child self-efficacy delta score, and parental factors measured at baseline.

	Parent support	Parent model	Parent resource	SedR	Ada	Pred	Enjoy	Intrin	Exter	Amot	Intro	Ident
6 weeks												
MVPA	0.107	0.179	0.139	-0.005	0.060	-0.005	0.244	0.206	-0.056	-0.190	0.109	0.125
Light PA	-0.010	0.169	0.065	-0.007	-0.057	0.005	0.032	0.055	-0.060	0.045	-0.037	-0.007
Sed Time	0.175	0.214	0.098	-0.018	-0.095	0.325*	-0.029	-0.021	-0.101	-0.010	-0.049	-0.176
Bouts Light PA	-0.052	0.195	0.021	-0.086	-0.018	-0.021	-0.083	0.009	0.002	-0.160	-0.245	0.209
Bouts MVPA	0.062	0.127	0.044	-0.053	-0.045	-0.055	0.226	0.172	0.058	-0.112	0.119	0.127
10 weeks												
MVPA	-0.077	0.021	0.164	-0.051	0.197	0.028	-0.006	0.295	0.045	-0.142	-0.316	-0.113
Light PA	0.168	0.223	0.368*	0.220	-0.120	0.171	-0.159	0.139	0.113	-0.052	-0.182	-0.181
Sed Time	-0.053	-0.002	0.035	0.099	0.008	0.138	0.249	-0.138	-0.257	0.060	0.067	0.017
Bouts Light PA	0.103	-0.101	0.341*	0.357*	-0.077	-0.214	0.430**	0.111	0.141	0.482**	-0.033	-0.011
Bouts MVPA	0.128	0.089	0.223	0.068	0.122	0.058	0.0104	0.385*	0.011	0.038	-0.156	-0.080

*p≤ 0.05;

**p<0.01

Parent support = Parent support of child’s PA (baseline)

Parent model = Parent role modeling PA for child (baseline)

Parent resource = parent uses public PA offerings (baseline)

SedR = Parent restricts child’s sedentary activity (baseline)

Ada = child’s perceived adequacy / competence in PA (delta)

Pred = child’s predilection towards PA vs sedentary activities (delta)

Enjoy = child’s enjoyment of PA (delta)

Intrin = child’s intrinsic motivation towards PA (delta)

Exter = child’s external motivation towards PA (delta)

Amot = child’s amotivation towards PA (delta)

Intro = child’s introjected motivation towards PA (delta)

**Table 5:** Univariate correlations of AVG, SVG, and TAP recall delta scores with parent scores and child self-efficacy.

	Logistic	Model	Resource	SedR	Ada	Pred	Enjoy	Intrin	Exter	Amot	Intro	Ident
*6 weeks*												
AVG	0.041	-0.023	-0.090	-0.115	0.044	0.142	-0.074	0.299*	0.202	-0.096	0.301*	.247
SVG	0.238	0.238	0.382**	0.353*	-0.079	0.079	-0.267	0.010	-0.078	0.081	0.056	0.103
TAP	0.184	0.106	0.069	0.043	-0.145	-0.009	-0.010	0.273	0.156	0.0003	0.225	0.333*
*10 weeks*												
AVG	-0.045	0.115	-0.096	0.024	-0.012	0.136	-0.136	0.214	0.337*	-0.216	0.238	0.219
SVG	0.189	0.341*	0.353*	0.373*	-0.144	-0.006	-0.129	-0.009	-0.061	0.065	-0.141	-0.058
TAP	0.399**	0.144	0.202	0.070	-0.242	-0.025	-0.075	0.085	0.078	0.012	0.139	0.050

*p≤0.05;

**p<0.01

Logistic = Parent logistic support of child’s PA (baseline)

Model = Parent role modeling PA for child (baseline)

Resource = parent uses public PA offerings (baseline)

SedR = Parent restricts child’s sedentary activity (baseline)

Ada = child’s perceived adequacy / competence in PA (delta)

Pred = child’s predilection towards PA vs sedentary activities (delta)

Enjoy = child’s enjoyment of PA (delta)

Intrin = child’s intrinsic motivation towards PA, AVG or SVG (delta)

Exter = child’s external motivation towards PA, AVG or SVG (delta)

Amot = child’s amotivation towards PA, AVG, or SVG (delta)

Intro = child’s introjected motivation towards PA, AVG or SVG (delta)

Ident = child’s identified motivation towards PA, AVG or SVG (delta)

## Discussion

Overall results indicated that exposure to AVG did not increase objectively measured MVPA or TAP. Light PA decreased over time while sedentary time increased, although none of the sedentary activities measured, including SVG play, showed increases. The present study extends previous research in this area by demonstrating that AVG play did not produce the unwanted effect of increasing time engaged in SVG or screen-based devices. In fact, AVG play was associated with a reduction in SVG time.; However, total sedentary time did not decrease, perhaps because SVG time was substituted with other sedentary behaviors, such as homework or reading. AVG play was also not an effective gateway for promoting children’s traditional active play or total physical activity. Based on previous work in adolescents that demonstrated AVG play was associated with SVG play [28] we hypothesized that access to AVG in children who do not usually play SVG could result in increased SVG play. However, encouragingly, rather than increase following AVG exposure, time spent in SVG play declined during the intervention and remained depressed during washout. In previous work, when adolescents who did habitually play SVG were exposed to AVG their SVG time also decreased compared to a wait-list control group [51]. While the authors noted that social desirability may have impacted the results [51], the current results support that the change observed in SVG was due in part to the AVG treatment. Thus, the data presented here indicate that children’s exposure to AVG reduces time spent playing SVG and previously published data from this cohort demonstrated that AVG play reduced the motivation to play SVG relative to AVG [29], arguing that both time spent in and motivation towards SVG play decreases when children play AVG.

Though SVG play time was reduced, total sedentary time increased by 22 minutes per day and light PA decreased by approximately 18 minutes per day. Which sedentary activities the children were substituting for light activity is difficult to ascertain, as the most common sedentary activities; television, computer, hobbies, or social activities did not change over time. Children also engage in non-leisure light activity, such as chores and accompanying parents on errands, and non-leisure sedentary activity, such as homework, practicing musical instruments, and traveling by car. Because children have less choice in how much time is spent in these non-leisure activities, this study chose not to focus upon such activities. However, changes in these activities may easily explain the time effects found for sedentary and light PA. Furthermore, even among leisure time activities, not all sedentary behavior is negative [52]. For example, reading and practicing musical instruments [53] have positive health benefits [54], while others such as television are associated with increased obesity and cardiometabolic risk [55]. PA interventions must take care to selectively disrupt only certain types of sedentary activity.

Overall, the present results demonstrate that AVG play does not increase overall PA amounts in children. These results are largely consistent with previous results from this cohort showing that AVG play does not increase motivation towards traditional active play (TAP) [29] or overall PA [18]. Fortunately, neither objectively measured MVPA, both in minutes and number of bouts, nor time spent in TAP decreased as a result of AVG play. AVG play did not displace time in TAP, perhaps because this study population spent very little time in TAP to begin with. Time spent in AVG play reflected the arc of the study: low at baseline, greater post-intervention, and low again at 10 weeks. Seasonal variations and the academic calendar did not alter this trajectory. This may reflect the overall lack of interest in PA (both TAP and AVG play) of the children that participated in this study, as most stopped playing AVG at 10 weeks. The present results are consistent with previous findings that most PA interventions for children produce small or negligible increases in PA [56]. Future research should continue to investigate both the barriers and facilitators of physical activity in children.

The questionnaire-based data sheds some light on reasons for barriers and facilitators of physical and sedentary activity change. The most consistent associations across both 6 and 10 weeks were between SVG and parental influence. Children’s time spent in SVG play was positively associated with parental restriction of sedentary activities and parents using public resources, such as parks, to help their children be physically active. A few possibilities exist. Regarding the negative association with parents use of public resources, parents who perceive that their children are too sedentary may be more likely to take their children to venues where they can be physically active. When reinforcing activities such as SVG are restricted, they may become more desirable [57]. This would be consistent with research demonstrating that when parents place restriction on children’s diets, children tend to eat more when restrictions are lifted [58]. However, others have demonstrated that children whose parents’ place restrictions on their TV viewing, watch less TV compared to more permissive parents [59]. For the duration of this study, parents were instructed to not put restrictions on their children playing SVG. During this unrestricted period, SVG play declined. This may be due to parents lifting the restriction or the sample of children finding SVG less appealing than other activities. If so, it implies that parents may be able to decrease the appeal of SVG play may be decreased both by removing the appeal of the “forbidden fruit” and making alternative activities more appealing.

The sole factor associated with increased MVPA bouts was changes in intrinsic regulation. Intrinsic regulation, or engaging in an activity for its own sake, has been consistently associated with PA in adults [60] and children [61, 62]. TAP was associated at 10 weeks with parental logistic support, such that children whose parents provided help such as driving them to places where they can be active, were more likely to engage in TAP. Other studies have found logistic support to be predictive of MVPA [63]. The current study purposefully selected for low-active children who were not involved in sports teams or organized activities. This subscale referenced sports teams specifically; however, depending on the child’s inclinations, parents may also provide logistic support for non-sport activities such as theatre club or Girl/Boy Scouts, which would provide a mixture of moderate, light, and sedentary activities and may displace sedentary screen time.

A few limits must be noted. It could be argued that declines in SVG resulted from loss of interest in their SVG system over time. However, it must be noted that at baseline, the participants spent an average of 49 minutes/day in SVG play, which decreased to 28 minutes/day during the AVG intervention and remained low following during washout, which suggests a sustained change from participants’ pre-intervention behavior. This finding would have been strengthened with the addition of a control group that did not receive AVG. Furthermore, our study was of limited duration; long-term PA reinforcement over the course of months or years is necessary for establishing life-long healthy habits [64]. Because study participants did not play AVG at baseline, the novelty of the games may have impacted results. It is possible that over time SVG play may have increased to baseline levels. Future studies with longer time-frames are needed, preferably also with a larger, more diverse sample. Future studies may also wish to investigate is whether the reduced time spent in sedentary screen-based activities translates into improved health-related outcomes. This may be especially so for youth with overweight or obesity. Recent research indicates while youth with obesity expended more energy overall in AVG play than youth with healthy weight, they engaged in less vigorous physical activity and less lower-body movements [17]. Though youth with greater body mass have greater energy expenditure [65, 66], differing activity patterns may ultimately attenuate AVG’s positive effects. More likely, reductions in sedentary screen activities would promote maintenance of a healthy body weight long-term by reducing energy intake [67] via snacks consumed during screen-time.

Correlations, while significant, tended to be weak. However, this is not unusual for psychological research and may instead indicate a complex interplay of multiple interacting relationships, for future research to examine. Also worthy of further study is why certain associations were significant at 6 weeks but not 10 weeks and vice versa. For example, AVG play was associated with changes in intrinsic motivation at 6 weeks but changes in external motivation at 10 weeks, perhaps indicating that they were initially driven by game novelty and later by study requirements. Whether these motivations would shift again after study requirements were lifted and children could engage in AVG play as they wished is unknown. Another noteworthy example is that changes in bouts of light PA at 10 weeks were associated with changes in several factors at 10 weeks, including parent provision of resources, restriction of sedentary activities, enjoyment of PA, and amotivation for PA, though these associations were not significant at 6 weeks. These results may reflect motivations which take longer to develop as formerly sedentary children become active. Also noteworthy is that the associations explored in these analyses were delta scores. It is possible that, for example, intrinsic and introjected motivation for AVG increased from baseline to 6 weeks and then remained high at 10 weeks, which manifested as no changes from 6 to 10 weeks.

Because inactive children were enrolled in this study, MVPA and TAP may have been low enough at baseline that a floor effect prevented post-intervention decreases. Likewise, children may have already been using screen-based devices to such an extent as to create a post-intervention ceiling effect. Furthermore, these results may not generalize to other age-groups, including younger children and adults. Although traditional PA results in greater energy expenditure, adults rate AVG as more enjoyable [68]. Given the well-known decrease in PA during the adolescent years, future studies may wish to investigate the ability of AVG to increase PA reinforcement during this developmental phase. Our results may also vary based upon the types of games offered, including both video game types and TAP. It is possible that children would have responded differently had their preferred game/sport or a team-based context been offered. Furthermore, a wide variety of AVG systems and games are currently on the market. Energy expenditure, enjoyment, and other characteristics relevant for continued long-term usage vary between games and systems [69–71], which suggests results from a study utilizing Xbox Kinect^™^ may not generalize to other system or games. Another limit is that the act of self-monitoring their PA, both in TAP and AVG, may have influenced children’s choices to be active or sedentary. Self-monitoring is often used as a weight loss technique in adults and may be an effective way to increase dietary or exercise compliance [72]. While the data, both via actigraph and self-report, does not show an upward trend of PA, it is possible that PA was greater at all time-points due to study monitoring.

In summary, this study is the first to specifically examine the impact of one type of screen-based play, AVG, upon a related but separate type of screen-based play, SVG. Results suggest that rather than synergistically increasing one another, increased AVG play leads to children decreasing their time in SVG play. AVG play also does not increase time spent in other screen-based activities. AVG play did not act as gateway toward time spent in TAP. About 20 minutes/day of light PA was substituted with sedentary time post-washout. However, the specific activity which increased could not be identified and may represent time in non-screen, non-leisure pursuits.

## Supporting information

S1 File Study protocol. This is an a priori protocol of study methods.
(DOCX)

S1 Checklist TREND checklist. This is a summary of TREND reporting guidelines.
(PDF)
