# Supplementary material for: The influence of active video game play upon physical activity and screen-based activities in sedentary children
Source: PLoS One. 2022 Jun 14;17(6):e0269057. doi: 10.1371/journal.pone.0269057 (PMC9197033; doi:10.1371/journal.pone.0269057)
Supplement: S1 File — This is an a priori protocol of study methods. (DOCX) [file pone.0269057.s001.docx]

**The Active Games Study: Increasing the Attractiveness of Active Video Game Play for Youth**

**Principal Investigator:** Kelsey Ufholz, Ph.D., James Roemmich, Ph.D.,

**A. Background and Significance/Relevance**

Childhood obesity has rapidly increased to reach epidemic proportions. As of 2012, nearly one third of children in the United States can be classified as overweight or obese, raising concern for a number of both short and long-term health consequences (CDC, 2015; Ogden et al., 2014). Increased childhood weight has been linked to leisure time sedentary activity (Strasburger, Jordan & Donnerstein, 2012). Therefore, reducing children’s sedentary activity and encouraging physical activity and have become major public health goals.

The majority of sedentary time takes the form of screen-based activities, including video games, television, computer games, phones, and tablets. In an effort to merge children’s favorite activities; sports and video games, and to help the player be more physically active (ACSM, 2016; Altamimi & Skinner, 2012), game developers have incorporated physical movements into game play. “Active video games” (AVGs) are now being studied as a method to increase children’s interest and participation in physical activity. Although research indicates that AVG playing may lead to energy expenditure comparable to light to moderate physical activity (Peng, Ling, & Crouse, 2011; Roemmich et al., 2012), children prefer sedentary video games to AVGS and often find such active games to be boring (Simons et al., 2015a; Simons et al., 2012). Children do not play AVGs as often or with the same intensity as traditional games/sports (Roemmich et al., 2012) and lose interest in them (Simons et al., 2015a); efforts must be made to increase the appeal of AVGs so they are competitive with sedentary video games.

Self-determination theory has been applied to the study of physical activity adoption and adherence (Teixeira et al., 2012). Self-determination theory posits that intrinsic motivation, or desire to engage in an activity because of enjoyment or personal fulfillment, might be increased through autonomy (Ryan & Deci, 2002). Past research has shown that increased autonomy via choice of how and when an activity is engaged in may help increase intrinsic motivation and therefore make an activity more appealing (Roemmich et al., 2012). Such increased internal motivation may help increase the relative reinforcing value (RRV) of AVGs, increasing the attractiveness of AVG games and making them a more competitive option compared to the already reinforcing sedentary games and thereby leading children to play active video games more often and more intensely than sedentary video games.

Ideally, increasing the RRV of AVG, would lead to increased motivation to play both the AVG and traditional forms of the activities upon which AVGs are based. Both AVG play and traditional games would lead to increased physical activity and greater energy expenditure. The magnitude of increase in energy expenditure would be greatest if AVG play served as gateway for traditional game play, as traditional forms of active games produce greater energy expenditure (Roemmich et al., 2012). However, AVGs’ may not serve as a gateway to increasing the motivation to engage in traditional games. Current research suggests that providing children with AVGs does not make them more active in general (Baranowski et al., 2012). There is no correlation between hours spent playing active video games and physical activity, but, rather, children who regularly play AVGs show a preference for other screen-based entertainment, such as television and sedentary video games (Simons et al., 2015b). Indeed, children often prefer sedentary video games to AVGs (Simons et al., 2012). It is therefore of concern that increasing children’s exposure to AVG may inadvertently increase their motivation to play sedentary video games rather than traditional sports/games thereby increasing sedentary time and decreasing overall energy expenditure. This tendency towards increased sedentary behavior may be further compounded by increased energy intake, especially in the form of energy-dense snacks and drinks (Pearson & Biddle, 2011). This study will examine whether the RRV of AVG may be increased by providing greater autonomy and whether increasing the RRV of AVG also increases the RRV of sedentary video games.

**B. Overall Objective and Specific Hypotheses to be tested**

The goal is to investigate how autonomy might be used to increase children’s intrinsic motivation towards active video games, active play, and/or sedentary activity. Specific hypotheses include:

**H1a (Primary):** High autonomy of AVG play will result in increased reinforcing value of AVGs relative to sedentary video games. Lower autonomy of AVG play will not show an increased reinforcing value of AVG relative to sedentary video games.

**H1b**: High autonomy of AVG play will increase intrinsic motivation to play AVGs relative to low-autonomy control condition.

**H1c:** High autonomy of AVG play will increase intrinsic motivation to engage in physical activity relative to low-autonomy control condition.

**H1d:** High autonomy of AVG play will result in more hours of AVG play during the follow-up phase relative to the low-autonomy control condition.

**H1e:** High autonomy of AVG play will increase usual physical activity during the follow-up phase relative to the low-autonomy control condition.

**H1f:** High autonomy of AVG play will result in increased reinforcing value of AVGs relative to traditional active games.

**H2a:** Increased reinforcing value of AVGs will result in increased reinforcing value of sedentary video games.

**H2b:** Increased reinforcing value of AVGs will result in increased hours/week spent playing sedentary video games.

**H2c**: Increased reinforcing value of AVGs will result in increased time spent in other screen-based sedentary activities (television, computer games, tablets/cell phones).

**H2d:** Increased time spent in screen-based sedentary activities will result in increased sweetened beverage/snack consumption.

**C. Methods**

**i. Participants and Screening:**

Participants for this study will consist of healthy sedentary children (ages 8-12 y). Sedentary behavior shall be operationalized, similar to previous studies (Epstein et al., 2011; Tremblay et al., 2011), as engaging in at least 14 hours of screen-based leisure time sedentary behavior (sedentary video games, television, computer games, cell phone/tablet) per week. Both non-overweight and overweight children (5^th^-95th BMI percentile) will be included. Initial screening will take place using a Survey Monkey based questionnaire. Entry criteria will include not currently trying to lose weight, no medical conditions which might impede physical activity, not engaging in leisure time moderate to vigorous physical activity more than three times per week for one hour at a time, not currently or previously owning an active video game system, not playing an active video game more than one-half hour per week, willingness to adhere to study treatments and measurement schedules, and access to a cellular phone to complete activity recall questionnaires Parents will provide informed consent and children will provide written assent before participation. The study will be approved by the University of North Dakota Institutional Review Board.

**ii. Design:**

The overall design will be a 2 group factorial design with autonomy condition treated as a between subject variable (high autonomy, low autonomy). Participants will be randomly assigned to a high autonomy group or a low-autonomy group. Time (baseline, end of 6 week intervention, 4 week follow-up) will be treated as a within-subject variable. Participants will be blocked according to Taves minimization on gender (male vs female), BMI (above 85^th^ percentile vs. below 85^th^ percentile), and baseline liking of AVG (7 and lower vs. 8-10 on 10 point Likert scale).

**iii. Procedures:**

*Screening and Consent Visit:* Initial screening will take place online. The first in-person session will include a review of answers to the online screening questions (Appendix 1), study procedures, informed consent/assent and baseline measurements. After providing consent/assent, parents will be asked to fill-out a basic demographics questionnaire (Appendix 2) and health history questionnaire (Appendix 3) regarding their child. Youth will be measured for height and weight and the parents and children will work together to complete the first of 4 Survey Monkey-based 24 hour recalls (Appendix 4) assessing baseline time that the child spent in physical activity, sedentary video games, and other screen-based sedentary activities. Baseline physical activity and sedentary behavior will be further assessed by having the participants wear an activity monitor (ActiGraph WGT3X-BT) for seven days and fill out a 24 hour activity recall on three more days, including a weekend day. Using questionnaires, children will be measured for their exercise self-efficacy (Appendix 5), intrinsic and extrinsic motivation for exercise (Appendix 6), intrinsic and extrinsic motivation for AVG (Appendix 7), intrinsic and extrinsic motivation for sedentary video games (Appendix 8), and snack/ sweetened beverage consumption (Appendix 9). Parents will also be asked to complete a series of questionnaires measuring their support of their child’s physical activities (Appendix10), and self-efficacy for their own exercise habits (Appendix 11). Parents will be permitted to assist their child completing the surveys if necessary.

*Baseline RRV Assessments:* RRV assessments will take place in the Grand Forks Human Nutrition Research Center (GFHNRC) laboratory space at Choice Health and Fitness Center. On separate days children will be assessed for their relative reinforcing value of active to sedentary video games (RRV_video games_) and RRV of active video games to traditional active games (RRV_active games_). Order of these tests will be counterbalanced among participants. Participants will be asked to sample and rate their liking of four active video games (Appendix 12), four inactive video games (Appendix 13), and four traditional games/sports (Appendix 14). Children will be permitted to briefly sample games with which they are not familiar. Following the RRV task, children will be permitted to play the games corresponding to time earned on the task. Active and sedentary video games shall be played at Choice Health and Fitness spaces. Participants and their parent will complete a 24 hour recall assessing baseline time spent in physical activity, sedentary video games, and other screen-based sedentary activities at the end of one of these visits. Parents and children will complete the 3^rd^ and 4^th^ of the 4 recalls during weekend days at home using SurveyMonkey.

*Week 1 and 3 Assessments:* Children will wear an activity monitor for 7 consecutive days in the same manner as at baseline assessment. Participating parents and children will also be asked to fill-out the 24 hour activity recall on 4 days, including at least one weekend day.

*End of-intervention (Week 6) Assessments:* Outcome assessments will take place during and just after the last week of the 6 week intervention. Post-intervention anthropometry, questionnaire, RRV, and physical activity testing will be identical to screening/baseline assessments.

*4 Week (Week 10) Post-Intervention Follow-up Assessments and Debriefing:* At 4 weeks following the end of the intervention, participants will complete their final assessment, to determine whether intervention effects on videogame reinforcement, AVG use, physical activity reinforcement, physical activity, and sedentary behavior have been maintained for at least 4 weeks. The assessments will be identical to screening/baseline. Following the assessment, participants will be debriefed, asked for intervention feedback (Appendix 15 & 16), thanked for their time, and compensated.

**Table 1. Testing Schedule**

|  | Screen | Baseline | Wk 1 | Wk 3 | End-Intervention  (Wk 6) | Follow-up  (Wk 10) |
| --- | --- | --- | --- | --- | --- | --- |
| Height | X |  |  |  | X | X |
| Weight | X |  |  |  | X | X |
| Demographic Questionnaires | X |  |  |  |  |  |
| PA/Sed Questionnaires | X | X | X | X | X | X |
| BSQ |  | X | X | X | X | X |
| RRV_video games_ |  | X |  |  | X | X |
| RRV_active games_ |  | X |  |  | X | X |
| Physical activity monitor |  | X | X | X | X | X |
| Debriefing |  |  |  |  |  | X |

**iv: Intervention:**

Participants will be randomly assigned to a high or low-autonomy group. Children in each group will be given an active video game system to use while enrolled in the study, and active video game DVDs requiring full body movements to operate. To evaluate whether AVG play impacts their motivation to engage in sedentary screen-based activities, children will also be given sedentary video games which can be operated using only hand and finger movement. To better simulate naturalistic conditions, the intervention will take place in participants’ homes. After both baseline RRV_activite games_ and RRV_video games_ have been assessed the intervention will commence. A researcher will visit the home at baseline to explain the intervention, set-up the AVG system, train the child and a parent on use of the system, and provide the activity monitor that the child will wear throughout the first, third, and sixth week of the intervention to track physical activity and sedentary behavior. The researcher will also provide a log and ask the child to record the date, duration of play, which game was played, and with whom as a check of study fidelity (Appendix 17). The researcher will stress to the child and parent the importance of following the research protocol, including wearing the activity monitor and recording AVG/video game play.

Children in the high autonomy group will be given two AVG games of their choice. Children in the low autonomy group will be given their most-liked game from the previous baseline RRV assessment. After two weeks, a researcher will return to the home to monitor progress and exchange the AVG and sedentary games. At each exchange, children in the high autonomy group will be allowed to select their next game, while children in the low autonomy group will receive a pre-determined game.

To facilitate the relatedness component of self-determination theory, children (both groups) will be encouraged to play the games with others, such as siblings, friends, or parents, and record with whom they played in the provided log. In the high autonomy condition, the children will be instructed to play the AVGs three times per week for a minimum of 20 minutes. Children in the low autonomy condition will be instructed that they must play three times per week for 20-40 minutes. Neither group will be given a specified time minimum or maximum minutes of play for sedentary video games. Additionally, children will not be told to play AVGs at any particular intensity, merely to record when and for how long they played the game, and to wear their activity monitors on the appropriate days.

Both groups will be given their highest liked sedentary video game from the baseline intervention. Parents will be discouraged from placing time limits on their children’s sedentary video game activities or taking away videogames as punishment for the duration of the study period, although parents need not make this known to the child.

*Post-Intervention Period:* During the four week post-intervention period, children will be provided with two AVGs and two sedentary games of their choice and told to play for however long and in whatever fashion they choose. Children will be asked to continue recording their play in the activity log. Parents will be told to not purchase or provide other AVGs or sedentary video games for their children during the intervention phase of the study, but they will be able to provide additional videogames during the four week post-intervention assessment. If additional video games are purchased, participants will be asked to report which games (active or sedentary) were bought and how much time was spent playing them. If there are already AVGs or sedentary video games in the household, participants will be asked to not play them until the end of the 6 week intervention, but can play them during the post-intervention period.

**v: Measurements:**

**Height and Weight.** Body weight will be measured via a Tanita scale after voiding. Participants will wear t-shirts, shorts and no shoes during measurement. Height will be measured using a stadiometer. BMI percentile will be calculated based upon growth curves for each participant’s age and gender (Kuczmarski et al., 2000).

**Active Video Game (AVG) Reinforcement Task.** Participants will be given a list with 4 AVGs and 4 age-appropriate sedentary video games. Participants will sample and then rate their liking for each game on a scale of 1-10, with greater scores indicating a greater preference for that game. The highest liked active and sedentary videogames will be made available during the testing session. Participants will then play a slot-machine style game in which mouse clicks earn them time towards their most-liked game. Two computers will be set up, one for sedentary and one for active video games. Participants will be permitted to move freely between the computers.

During the game, three shapes of different colors will appear upon the screen. The participant will click upon the shapes in an attempt to make the shapes/colors match. Each match will earn one point, with 5 points earned at a particular schedule translating to minutes in each activity (in five minute intervals). Participants will be permitted to earn as much time with each game as they choose. The RRV of each game will be assessed by total responses and by maximum level completed (Pmax) which a participant is willing to make in order to earn minutes of play in their desired activity. Reinforcing value is measured using progressive ratio schedules of reinforcement. At first, points are delivered after every 4 presses, but then the schedule of reinforcement doubles (4, 8, 16, 32, […] 1024) each time 5 points are earned.

Participants will be permitted to earn points until they have accumulated the time they wish or they no longer wish to engage in the task. Before commencement of the task, the researcher will explain the task and demonstrate with a practice round. The participant will complete the task alone with the researcher monitoring their progress via computer and audio feed in an adjacent room. After task completion, the participant will be permitted to complete the AVGs and/or sedentary video game with the participant playing against the game rather than against an opponent. While the child is completing the AVG reinforcement task, the parent will waiting in an adjacent room, to avoid influencing the child’s responses.

**Physical Activity (PA) Reinforcement Task.** The physical activity reinforcement task will follow a schedule identical to the AVG reinforcement task. The task will also follow a procedure identical to the AVG reinforcement task, with the exception that the two workstations will allow the participant to earn time towards either AVGs or a traditional game/sport. The AVG and traditional sport/games will be supervised by a researcher and will include games comparable to the AVG games. The traditional active game will be completed indoors at the Choice Fitness Center. Children will have the options of playing traditional games/activities such as hockey (with a puck, stick and net), soccer (with a ball, cones, and net), racquetball (with a racquet and ball), and basketball (with a ball and net).

**Usual Physical activity.** PA will be assessed with the ActiGraph WGT3X-BT monitor. The device has a 3-axis accelerometer to collect measures of raw acceleration, and PA amount and intensity, and sedentary time. The ActiGraph has been extensively used in research studies and validated for use in children ages 8-12 (Ekelund et al., 2001; Puyau, 2002; Trost et al., 1998) Children will be instructed to put the ActiGraph on upon waking and only take it off while sleeping or in water-based activities. Parents will be trained regarding the importance that their child wears the ActiGraph at least 10 hours/day (standard to ensure validity) including the times that they play AVG for 7 consecutive days during the indicated study periods. Parents will receive written instructions on use, including appropriate care and placement on the right iliac crest (a belt is provided) (Appendix 18) (Patterson et al., 1993). The ActiGraph will be initialized for 5 second epochs and the data downloaded using the ActiLife software and stored in a “dat” file. Then, a customized Systat code will be used to identify spurious data of >16,000-20,000 counts, negative counts, and strings of identical counts. It will score sequences of 20 + minutes of consecutive zero counts as non-wear time. The total amount of PA from the activity monitor will be expressed as the average of total counts per minute of time worn. The main outcome variables will be mean counts/minute an index of mean total PA and time spent in moderate to vigorous PA (MVPA). The ActiGraph data will be converted to 15-sec epochs and MVPA determined using the count thresholds developed by Evenson et al. (2008; Trost et al., 2011).

**Physical Activity Mode and Sedentary Activity.** Physical activity and sedentary activities will be primarily assessed using the accelerometers. However, accelerometers do not provide detail of the mode of physical activity or sedentary behavior. As an adjunct, children will complete a 24 hour recall based upon the procedures described in Simons and colleagues (2015). Children will be asked to indicate for three time periods (awakening to noon, noon to 4 PM, and 4 PM until bedtime) how much time they had spent in a predetermined list of activities including active video games, sedentary video games, other screen-based sedentary activities (TV, computer, IPad), non-screen based sedentary activities (homework, music/art lessons), physical activity (sports, physical education classes), and other physical activity (chores, walking to school) (see Appendix 4). Categories will be slightly modified from the original study to make them age-appropriate. Participants will complete the recall on 4 days, including two school-days and two weekend days. The participating parent will be sent text and/or email links to the recall on Survey Monkey, so that they may fill out the recall at the end of each day. Parents will be encouraged to work with their child to complete the recall.

**Beverage and Snack Consumption.** To measure non-meal snacking during playtime or games, children will be administered the Beverage and Snack Questionnaire (BSQ) (Neuhouser et al., 2009). This tool consists of 19 questions designed to measure consumption of common sugar-sweetened beverages and snack foods, both in school and at home. The tool was designed for school-aged children (mean age = 12.7). Validation studies have found this measure to have good reliability and validity (Neuhouser et al., 2009) (see Appendix 9).

***Mediators and Moderators***

**Exercise Self-Efficacy.** Self-efficacy will be measured using the *Children’s Self-Perceptions of Adequacy in and Predilection of Physical Activity Scale (CSAPPA)*. This scale measures child’s beliefs about their ability to perform well in physical activities such as physical education classes and sports teams (adequacy), their likelihood to choose physical activity over a sedentary activity (predilection), and enjoyment of physical activity. The scale consists of 20 items which children rate as “really true for me” or “sort of true for me” (Hayes, 1992). The scale was designed for use with children ages 8-16 and has been successfully used experiments with similar age groups (Roemmich et al., 2012). The scale has been shown to have adequate test-retest reliability as well as predictive validity (Hayes, 1992) (see Appendix 5).

**Motivation for Exercise.** Intrinsic and extrinsic motivation for exercise will be measured with the *Behavioral Regulations in Exercise Questionnaire, 2^nd^ edition* (BREQ-2). The original BREQ was developed as a measure the self-determination theory’s continuum of external vs. internal motivation for exercise (Mullan, Markland & Ingledew, 1997). The original version was developed for use with adults and examined four subscales: external regulation, introjected regulation, identified regulation and intrinsic regulation. The BREQ-2 includes an additional subscale measuring Amotivation (Wilson et al., 2006). The BREQ has good psychometric properties with children ages 7-11 (Sebire et al., 2013) and the BREQ-2 has been utilized successfully with children ages 8-11 years (Saavedra et al., 2014). The questions may be slightly modified to make them more easily comprehensible to children (see Appendix 6).

**Motivation for Active Video Games (AVGs).** Questions from the BREQ-2 (Wilson et al., 2006) will be modified so that the question refers to motivation towards AVGs rather than physical activity. While not specifically designed for alternate behaviors, variations of the BREQ-2 have been successfully used to measure motivations for non-exercise behaviors such as children’s oral health (Toutouni et al., 2014). As validation, this measure will be examined for internal validity via Cronbach’s alpha and criterion validity via correlations with RRV_video games_ and minutes spent playing AVGs (see Appendix 7).

**Motivation for Sedentary Behavior**. Questions from the BREQ-2 (Wilson et al., 2006) will be modified so that the question refers to motivation towards sedentary behavior (non-active video games, television, computers etc.) rather than physical activity. Similar to motivation for AVGs, this questionnaire will be validated via Cronbach’s alpha, RRV correlations, and minutes spent in sedentary activity (see Appendix 8).

**Physical activity-related parenting behaviors.** Parents will fill in The Activity Support Scale for Multiple Groups (ACTS-MG) developed by Davison (2011). The ASSMG includes 12 items, 3 each in: Logistic Support (parents making provisions enabling their child to be physically active), Explicit Modeling (parents using their own behavior to encourage their child to be active), Use of Community Resources (make use of community resources to get their child to be active) and Restricting Access to Sedentary Activities. Cronbach’s alpha ranges from 0.69 to 0.88. Items are measured on a 4 point scale (0 = strongly disagree; 4= strongly agree) with higher scores showing greater parental support for physical activity. The scale has good overall factorial validity and internal consistency (Davidson et al., 2011) (see Appendix 10).

**Parent self-efficacy for exercise.** The Barriers Self-efficacy Scale (BARSE) (McAuley, 1992). For the BARSE they rate how confident they are that they could motivate themselves to exercise regularly, for at least three months, despite common barriers. The scale consists of 13 items measured on as 10 point percentages (0 = not at all confident; 100 = highly confident). Items are summed with higher scores indicating greater self-efficacy. Scores predict exercise behavior (McAuley, 1991). The scale has good internal consistency and validity (McAuley et al., 2010) (see Appendix 11).

**D. Statistical Analysis Plan**

*Power analysis and sample size:*

Prior research has shown an effect size of approximately eta squared (h^2^) = 0.45 and a mean difference between high (M = 41.4) vs low autonomy (M = 22.5 minutes of play) conditions (difference = 18.9 minutes) (Roemmich et al., 2012). Based upon these and conservative estimates from Cohen’s guidelines for multivariate effects (Cohen, 1992), estimates of required sample size to achieve power exceeding the 1 - β = .90 level were run and a sample size of N = 25 participants per autonomy condition was recommended.

Independent variables will be the study condition of high vs. low autonomy (categorical variable) and time (categorical). Dependent variables will consist of total scores in RRV_video games,_ total scores in RRV_active games_, total weekly minutes of physical activity, total weekly minutes of AVG play, total weekly minutes of sedentary video game play, total weekly minutes of other screen-based sedentary activity, total weekly minutes of non-screen based sedentary activities, maximum level completed towards AVGs, maximum level completed towards sedentary video games, BMI/ weight loss, total energy expenditure, exercise self-efficacy, intrinsic motivation towards AVGs, intrinsic motivation towards exercise, intrinsic motivation towards sedentary video games, sweetened beverage/snack consumption (continuous variables). Participant exercise self-efficacy, parental exercise self-efficacy, and parental support for physical activity will be treated as moderating covariates (continuous).

*Preliminary Analyses:*

Preliminary data analysis will consist of all continuous data being examined for normality assumptions (skew, kurtosis, linearity, homogeneity of variance). Demographics will be tallied and basic descriptives including mean, median, maximum and minimum scores, and standard deviations calculated. For categorical variables, frequencies will be run. While significant attrition is not anticipated, participants failing to provide complete data will be compared to participants providing complete data in terms of baseline measures of age, gender, ethnicity, study condition, and baseline RRV of the key dependent variables via ANOVA tests for continuous variables and chi-square tests for categorical variables. Similar comparisons of baseline variables will be used to compare the high vs low autonomy groups to ensure that all possible confounds are evenly distributed at baseline. No significant differences between groups are anticipated. Bivariate correlations will also be run between the main study variables, both at baseline and at post-assessment. For each of the main questionnaires, internal validity of each total scale and subscale will be measured via Cronbach’s alpha.

*Major Analyses:*

Major analyses for Hypothesis 1 will take initially the form of a repeated measures analysis of covariance (ANCOVA). The between-subjects grouping variable will be high vs low autonomy. Outcome variables will take the form of means and standard deviations with 95% confidence intervals. Baseline variables, age, gender, and baseline enjoyment of AVGS will be included as control variables/ possible moderators. Moderating variables will include participant’s exercise self-efficacy, parental support for physical activity, and parental exercise self-efficacy. Moderating variables will be run separately. Each dependent variable shall be run separately.

Major analyses for Hypothesis 2 will take the form of multilevel regression equations. The main independent variable will be the changes in total reinforcement value (Pmax) of AVGs. Covariates will include age, gender, baseline enjoyment of sedentary video games, baseline time spent playing sedentary video games and other screen-based activities. The primary outcome variables will be maximum level earned (Pmax) towards sedentary video games and minutes of sedentary video games played per week. Once again, results will be considered statistically significant at α = .05. Covariates will be considered statistically significant as evidenced by significant β coefficients and significant increases in R^2^ values. Categorical covariates will be dummy-coded according to the procedure outlined in Cohen et al. (2003).

**E. Anticipated Results and Likely Impact**

There is anticipated to be a significant omnibus result. Specifically it is anticipated that high-autonomy participants will show greater increases in reinforcing values of AVGs relative to sedentary video games compared to low-autonomy participants, as evidenced by a greater mean on the RRV task (Hypothesis 1a). It is also anticipated that, compared to participants in the low autonomy condition, participants in the high autonomy condition will show greater intrinsic motivation towards AVGs (Hypothesis 1b) and greater intrinsic motivation towards exercise (Hypothesis 1c). It is further anticipated that participants in the high autonomy condition will play AVGs a comparatively greater number of minutes per week (Hypothesis 1d) compared to the low autonomy condition. It is also anticipated that participants in the high autonomy condition will increase minutes of usual physical activity per week to a greater extent than will participants in the low autonomy condition (Hypothesis 1e). It is also anticipated that participants in the high autonomy condition will show greater increases in reinforcing values of AVGs relative to traditional games/sports compared to low-autonomy participants, as evidenced by a greater mean on the RRV task (Hypothesis 1f). For hypothesis 2, a significant overall effect is also anticipated. Specifically it is anticipated that increases in the total reinforcing values of AVGs (Pmax) will significantly predict increases in the total reinforcing values of sedentary video games (Hypothesis 2a), increases in minutes/ week spent playing sedentary video games (Hypothesis 2b), and increases in minutes/week spent in other screen-based sedentary activities (Hypothesis 2c). Increased time spent in other screen-based sedentary activities is also anticipated to result in increased sweetened beverage/snack consumption (Hypothesis 2d).

**F. Human Participant Burden (Time, Discomfort, Inconvenience)**

**Table 2. Time to Complete Each Measurement**

|  | # Days Assessed | Min to complete/day |
| --- | --- | --- |
| Online screening | 1 | 10 |
| Informed Consent/Assent | 1 | 10 |
| Height | 3 | 5 |
| Weight | 3 | 5 |
| Demographics | 1 | 5 |
| 24 Hour PA recall | 20 | 10 |
| CSAPPA (Exercise self-efficacy) | 3 | 5 |
| BREQ-2 (exercise motivation | 3 | 5 |
| BREQ-2 (AVG motivation) | 3 | 5 |
| BREQ-2 (sedentary motivation) | 3 | 5 |
| BSQ | 5 | 5 |
| ACTS-MG | 1 | 5 |
| BARSE | 1 | 5 |
| RRV_video games_ | 3 | 60 |
| RRV_activity_ | 3 | 60 |
| Accelerometer | 21 | As necessary |
| Debriefing | 1 | 10 |

**G. Requested Participant Reimbursement Schedule**

Reimbursement for this study will take the form of a $435 check for the child and a $110 check for the parent or a family membership to Choice Fitness Center for 9 months ($600 value). Choice of the checks or family membership will be at the discretion of the parent. Participants who do not complete the study will receive a pro-rated amount based upon the number of weeks and assessments completed.

**H. Impact on Center Resources**

**i. Budget:**

**Table 3. Total Project Budget**

| Item |  | Cost | Item/  participant | Cost/  participant | Total cost  (estimated N = 50) |
| --- | --- | --- | --- | --- | --- |
| Recruitment |  | 2000 |  |  | 2000 |
| Screening |  |  | 1 |  |  |
| Transportation |  | 0 | 0 | 0 | 0 |
| Questionnaires |  |  |  |  |  |
|  | Weight | 0 | 3 | 0 | 0 |
|  | Height | 0 | 1 | 0 | 0 |
|  | Demographics | 0 | 1 | 0 | 0 |
|  | PA Recall | 0 | 16 | 0 | 0 |
|  | CSAPPA | 0 | 3 | 0 | 0 |
|  | BREQ-2 (exercise) | 0 | 3 | 0 | 0 |
|  | BREQ-2 (AVG) | 0 | 3 | 0 | 0 |
|  | BREQ-2 (sedentary) | 0 | 3 | 0 | 0 |
|  | BSQ | 150 | 3 | 0 | 150 |
|  | ACTS-MG | 0 | 1 | 0 | 0 |
|  | BARSE | 0 | 1 | 0 | 0 |
| Assessments |  |  |  |  |  |
|  | RRV_video games_ | 0 | 3 | 0 | 0 |
|  | RRV_activity_ | 0 | 3 | 0 | 0 |
| Child reimbursement |  |  |  | **$435** | **$21750** |
| Parent reimbursement |  |  |  | **$110** | **$5500** |
| **Grand Total** |  |  |  |  | **$27250** |

ii. Support Staff Time Needed:

*Recruitment:* 1-2 hours/ participant. Total = 50-100 hours

*Screening and Consenting*: 1-1.5 hours / participant. Total = 50-75 hours

*CARES Scheduling:* 2-3 hours per participant for set-up and maintenance. Total 100-150 hours

*Transportation:* 0 hours

*Anthropometry (height, weight):* 15 minutes direct participant time, 0.5 hours prep and clean-up, 0.5 hour data reduction and transfer Total = 62.5 hours

*Baseline Assessment Questionnaires:* 2-2.5 hours/participant direct time. 0.5 hour prep and clean-up, 0.5 hour data reduction. Total = 150-175 hours

*Baseline RRV_activity_:* 1 hour/ participant direct participant time, 0.5 hours prep and clean-up, 0.5 hour data reduction and transfer. Total = 100 hours

*Baseline RRV_video games_:* 1 hour/ participant direct participant time, 0. 5 hours prep and clean-up, 0.5 hour data reduction and transfer. Total = 100 hours

*Home Installation of Games:* 1 hour/ participant. Total = 50 hours

*2 and 4 Week Check-up:* 0.5 hour per check-up per participant. Total = 50 hours

*Post-intervention Assessment Questionnaires:* 2 hours per participant, 0.5 hour prep and clean-up, 0.5 hour data reduction. Total = 150 hours

*Post-intervention RRV_activity_:* 1 hour/ participant direct participant time, 0. 5 hours prep and clean-up, 0.5 hour data reduction and transfer. Total = 100 hours

*Post-intervention RRV_video games_:* 1 hour/ participant direct participant time, 0. 5 hours prep and clean-up, 0.5 hour data reduction and transfer. Total = 100 hours

*Debriefing and Compensation:* 1 hour/ participant. Total = 50 hours

*Statistics:* 24 hours estimated time for LuAnn Johnson.

*Information Technology:* 12 hours estimated time for Doreen Reindel-Rolshoven and ITS support staff.

iii: Facilities Needed: The project primarily utilize the Choice Health and Fitness Center laboratory for informed consent/assent, debriefing, the RRV tests, height/weight measurement, and the questionnaires. The intervention will take place in participants’ homes. Statistical consultation will be provided by LuAnn Johnson.

**I. Participant Recruitment Plan**.

This study will be advertised using various media. Advertisements will be placed in the Dakota Student, Grand Forks Herald, and UND Listserv, both for the general student body and for faculty (Appendix 19a). In addition printed brochures (Appendix 19b) and flyers (Appendix 19c) will be made available in places frequented by families, such as Choice Health and Fitness Center, The UND Child’s Learning Center, the YMCA, the children’s room in the public library, local grocery stores, community bulletin boards, local malls/shopping centers etc. Information about this study will also be made available on the GFHNRC website.

**J. Timetable.**

Because children have more free time and therefore different patterns of play and activity during the summer months, this study will take place during the school year. It is anticipated that this study will be approved by the Institutional Review Board no later than October 2016. Recruitment will commence shortly afterwards. The intervention will last 6 weeks, followed by a 4 week wash-out period. Data analysis is anticipated to take 2-4 weeks and report writing 1-2 months.

**References**

Altamimi, R. & Skinner, G. (2012). A survey of active video game literature. *International Journal of Computer and Information Technology, 1(1),* 2277-0764.

American College of Sport Medicine (2016). Exergaming. Public Information Brochures and Fact Sheets. Retrieved April 22, 2016.

Baranowski, T., Abdelsamad, D., Baranowski, J., O’Connor, T. M., Thompson, D., Barnett, A., Cerin, E., & Chen, T. (2012). Impact of an active video game on healthy children’s physical activity. *Pediatrics, 129(3),* e636-e642.

Centers for Disease Control and Prevention (2015, May 15). About childhood & teen BMI. Retrieved April 12, 2016 from http://www.cdc.gov/healthyweight/assessing/bmi/childrens_bmi/about_childrens_bmi.html

Centers for Disease Control and Prevention (2015, August 27). Childhood obesity facts. Retrieved March 31, 2016 from http://www.cdc.gov/healthyschools/obesity/facts.htm,..

Cohen, J. (1992). A power primer. *Psychological Bulletin, 112*, 155-159.

Cohen, J., Cohen, P., West, S. G., & Aiken, L. S. (2003). *Applied Multiple Regression/ Correlation Analysis for the Behavioral Sciences, 3^rd^ edition*. Mahwah, NJ: Lawrence Erlbaum Associates, Publishers.

Davison, K.K., Li, K., Baskin, M.L., Cox, T., & Affuso, O. (2011). Measuring parental support for children's physical activity in white and African American parents: the Activity Support Scale for Multiple Groups (ACTS-MG). *Preventive Medicine,* 52(1),39-43.

Ekelund, U., Sjöström, M., Yngve, A., Poortvliet, E., Nilsson, A., Froberg, K., Wedderkopp, N., & Westerterp, K. (2001). Physical activity assessed by activity monitor and doubly labeled water in children. *Medicine & Science in Sports & Exercise,* 33(2), 275-281.

Epstein, L. H., Roemmich, J. N., Cavanaugh, M. D., & Paluch, R. A. (2011). The motivation to be sedentary predicts weight change when sedentary behaviors are reduced. *International Journal of Behavioral Nutrition and Physical Activity, 8(13)*.

Everson, K. R., Catellier, D. J., Gill, K., Ondrak, K. S., & McMurray, R. G. (2008). Calibration of two objective measures of physical activity in children. *Journal of Sports Science, 26(14),* 1557-65.

Hay, J. A. (1992). Adequacy in and predilection for physical activity in children. *Clinical Journal of Sport Medicine, 2(3),* 192-201.

Kuczmarski, R. J., Ogden, C. L., Grummer-Strawn, L. M., Flegal, K. M., Guo, S. S., Wei, R., Mei, Z., Curtin, L. R., Roche, A. F., & Johnson, C. L. (2000). CDC growth charts for the United States: methods and development. National Center for Health Statistics. *Vital Health Statistics, 11(246)*.

McAuley, E. (1993). Self-efficacy and the maintenance of exercise participation in older adults. *Journal of Behavioral Medicine, 16,* 103-113.

McAuley, E. (1992). The role of efficacy cognitions in the prediction of exercise behavior in middle-aged adults. *Journal of Behavioral Medicine, 15(1),* 65-88.

McAuley, E., Mailey, E. L., Mullen, S. P., Szabo, A. N., Wójcicki, T. R., White, S. M., Goethe, N., Olson, E. A., & Kramer, A. F. (2010). Growth trajectories in exercise self-efficacy in older adults: influence of measure and initial status. *Health Psychology, 30(1),* 75-83.

Mullan, E., Markland, D., & Ingledew, D. K. (1997). A graded conceptualisation of self-determination in the regulation of exercise behaviour: development of a measure using confirmatory factor analytic procedures. *Personality and Individual Differences, 23(5),* 745-752.

Neuhouser, M. L., Lilley, S., Lund, A., & Johnson, D. B. (2009). Development and validation of a beverage and snack questionnaire for use in evaluation of school nutrition policies. *Journal of the American Diabetic Association, 109,* 1587-1592.

O’Connor, T. M., Chen, T., Baranowski, J., Thompson, D., & Baranowski, T. (2013). Physical activity and screen-media-related parenting practices have different associations with children’s objectively measured physical activity. *Childhood Obesity, 9(5),* 446-453.

Ogden, C. L., Carroll, M. D., Kit, B. K., & Flegal, K. M. (2014) Prevalence of childhood and adult obesity in the United States, 2011-2012. *Journal of the American Medical Association, 311(8),* 806-814.

Patterson, S. M., Krantz, D. S., Montgomery, L. C., Deuster, P. A., Hedges, S. M., & Nebel, L. M. (1993). Automated physical activity monitoring: validation and comparison with physiological and self-report measures. *Psychophysiology, 30(3),* 296-305.

Pearson, N. & Biddle, S. J. H. (2011). Sedentary behavior and dietary intake in children, adolescents, and adults: A systematic review. *American Journal of Preventive Medicine, 41(2),* 178-188.

Peng, W., Lin, J., & Crouse, J. (2011). Is playing exergames really exercising? A meta-analysis of energy expenditure in active video games. *Cyberpsychology, Behavior, and Social Networking, 14(11),* 681-688.

Roemmich J. N., Lambiase, M. J., McCarthy, T. F., Feda, D. M., & Kozlowski, K. F. (2012) Autonomy supportive environments and mastery as basic factors to motivate physical activity in children: a controlled laboratory study. *International Journal of Behavioral Nutrition and Physical Activity, 9,* 16.

Ryan, R. M. & Deci, E. L.(2002). Overview of self-determination theory: An organismic dialectical perspective. In E. L. Deci & R. M. Ryan (Eds.), *Handbook of Self-Determination Research*. Rochester, NY: University of Rochester Press, 3-33.

Saavedra, J. M., García-Hermoso, A., EscalanteY., & Domínguez, A. M. (2014). Self-determined motivation, physical exercise and diet in obese children: a three-year follow-up study. *International Journal of Clinical and Health Psychology, 14,* 195-201.

Sebire, S. J., Jago, R., Fox, K. R., Edwards, M. J., & Thompson, J. L. (2013). Testing a self-determination theory model of children’s physical activity motivation: a cross-sectional study. *International Journal of Behavioral Nutrition and Physical Activity, 10,* 111.

Simons, M., Brug, J., Chinapaw, M. J., de Boer, M., Seidell, J., & de Vet, E. (2015a) Replacing non-active video gaming by active video gaming to prevent excessive weight gain in adolescents. *PLOS One, 10(7),* e0126023.

Simons, M., Chinapaw, M. J., Brug, J., Seidell, J., & de Vet, E. (2015b). Associations between active video gaming and other energy-balance related behaviours in adolescents: a 24-hour recall diary study. *International Journal of Behavioral Nutrition and Physical Activity, 12(1),* 32.

Simons, M., de Vet, E., Hoornstra, S., Brug, J., Seidell, J., & Chinapaw, M. J. (2012). Adolescents’ views on active and non-active videogames: a focus group study. *Games for Health Journal, 1(3),* 1-8.

Strasburger, V.C., Jordan, A.B., & Donnerstein, E. (2012). Children, adolescents, and the media: health effects. *Pediatric Clinics of North America, 59,* 533-87.

Teixeira, P. J., Carraça, E. V., Markland, D., Silva, M. N., & Ryan, R. M. (2012). Exercise, physical activity, and self-determination theory: A systematic review. *International Journal of Behavioral Nutrition and Physical Activity, 9(1),* 78.

Toutounti, H., Ghofranipour, F., Akbarzadeh, A., & Zafarmand, A. H. (2014). Modification and validation of behavioral regulation in exercise questionnaire (Breq-2) for oral health care in young children. *Journal of Theory and Practice in Dental Public Health, 2(3-4),* 19-25.

Tremblay, M. S., LeBlanc, A. G., Kho, M. E., Saunders, T. J., Larouche, R., Colley, R. C., Goldfield, G., & Gorber, S. C. (2011). Systematic review of sedentary behavior and health indicators in school-aged children and youth. International Journal of Behavioral Nutrition and Physical Activity, 8, 98.

Trost, S. G., Loprinzi, P. D., Moore, R., & Pfeiffer, K. A. (2011). Comparison of accelerometer cut points for predicting activity intensity in youth. Medicine & Science in Sport & Exercise, 43(7),1360-1368.

Trost, S. G., Ward, D. S., Moorehead, S. M., Watson, P. D., Riner, W., & Burke, J. R. (1998). Validity of the computer science and applications (CSA) activity monitor in children. *Medicine & Science in Sports & Exercise, 30(4),* 629-633.

Wilson, P. M., Rodgers, W. M., Loitz, C.C., & Scime, G. (2006). “It’s who I am…really!” The importance of integrated regulation in exercise contexts. *Journal of Biobehavioral Research, 11,* 79-104.
